# Characterization of a dual media system for culturing primary normal and Fuchs endothelial corneal dystrophy (FECD) endothelial cells

**DOI:** 10.1371/journal.pone.0258006

**Published:** 2021-09-29

**Authors:** Tommy A. Rinkoski, Cindy K. Bahler, Johann M. Pacheco, Maya L. Khanna, David M. Holmes, Uttio Roy Chowdhury, Keith H. Baratz, Sanjay V. Patel, Leo J. Maguire, Eric D. Wieben, Michael P. Fautsch

**Affiliations:** Department of Ophthalmology, Mayo Clinic, Rochester, MN, United States of America; Cedars-Sinai Medical Center, UNITED STATES

## Abstract

Primary cultures of human corneal endothelial cells (HCECs) are an important model system for studying the pathophysiology of corneal endothelium. The purpose of this study was to identify and validate an optimal primary culture model of normal and Fuchs endothelial corneal dystrophy (FECD) endothelial cells by comparing cell morphology and marker expression under different media conditions to in vivo donor tissues. Primary and immortalized HCECs, isolated from normal and FECD donors, were cultured in proliferation media (Joyce, M4, Bartakova) alone or sequentially with maturation media (F99, Stabilization 1, M5). CD56, CD73 and CD166 expressions were quantified in confluent and matured cell lines by flow cytometry. HCECs that were allowed to proliferate in Joyce’s medium followed by maturation in low-mitogen containing media yielded cells with similar morphology to corneal endothelial tissues. Elevated expression of CD56 and CD166 and low expression of CD73 correlated with regular, hexagonal-like HCEC morphology. CD56:CD73 > 2.5 was most consistent with normal HCEC morphology and mimicked corneal endothelial tissue. Immortalization of normal HCECs by hTERT transduction showed morphology and CD56:CD73 ratios similar to parental cell lines. HCECs established from FECD donors showed reduced CD56:CD73 ratios compared to normal HCECs which coincided with reduced uniformity and regularity of cell monolayers. Overall, a dual media system with Joyce’s medium for proliferation and a low-mitogen media for maturation, provided normal cultures with regular, hexagonal-like cell morphologies consistent with corneal endothelial cells in vivo. CD56:CD73 expression ratio >2.5 was predictive of in vivo-like cellular morphology.

## Introduction

The corneal endothelium, a monolayer of cells on the inner surface of the cornea, is responsible for maintaining the deturgescence and transparency of corneal tissue by pumping fluid from the corneal stroma into the anterior chamber of the eye [1]. The cells of the endothelial monolayer are non-proliferating and arrested in the G1 phase of their cell cycle [2,3]. Changes to these cells via gene specific mutation and/or effects of environmental exposure can result in loss of normal function and development of pathologic conditions such as Fuchs endothelial corneal dystrophy (FECD) [4–7].

Detailed investigation of the underlying mechanisms associated with FECD has been impeded by difficulty in obtaining diseased tissue, relative acellularity of this tissue, and the inability to isolate sufficient quantities of DNA, RNA, or protein for assay. Because of these limitations, the most utilized model system to study human corneal endothelial cells is primary or immortalized monolayer cell culture. Primary human corneal endothelial cell (HCEC) lines have been established in culture through a variety of methods [8–13]. These include the use of a high mitotic proliferation media throughout the culture period or a dual media approach in which the first medium is rich in growth factors to promote cellular proliferation and the second medium contains reduced levels of mitogenic factors to encourage cells to mature into a hexagonal-like monolayer reminiscent of the honeycomb appearance of corneal endothelial tissue in vivo. The low-mitogen maturation medium is patterned after the in vivo anterior chamber environment where corneal endothelial cells are bathed in aqueous humor, a specialized fluid that contains only 1/70th the protein concentration in serum [14,15]. Culture methods using various maturation media have been used for producing primary HCECs [16,17]. Recently, HCECs grown using a dual media approach with low-mitogen maturation medium were shown to have more robust barrier and pump functions than HCECs grown in a higher mitogen medium [18].

HCECs in culture can show wide variability in their patterns of proliferation and morphology, ranging from a regular hexagonal-like endothelial appearance to markedly fibroblast-like characteristics. Some reports suggest that cell surface markers such as CD56 (neural cell adhesion molecule 1; NCAM1) and CD166 (activated leukocyte cell adhesion molecule; ALCAM) may be associated with the desired corneal endothelial morphology [19] when expressed by HCECs in vitro [17,20,21]. In contrast, increased expression of CD73 (ecto-5’-nucleotidase; NT5E) has been found to correlate with endothelial-mesenchymal transition and a fibroblast-like appearance in HCECs [21]. In order to use primary HCEC cultures as a robust in vitro model, it is imperative to standardize and optimize culture conditions that simulate cellular characteristics found in vivo. In the current study, we evaluated the effects of several culture conditions on HCEC morphology and performed correlative studies to link expression of CD (cluster of differentiation) marker phenotypes to desired in vivo-like cell morphology. The results provide correlative and predictive parameters to assess the degree of in vivo-like morphology of both primary and immortalized HCECs established from normal, as well as FECD corneal tissues.

## Methods

### HCEC culture

The use of human tissue for research was in compliance with the Mayo Clinic Institutional Review Board and followed the tenets of the Declaration of Helsinki. Patients with advanced FECD were enrolled into the Mayo Clinic Hereditary Eye Disease Study (IRB 06–007210) after informed consent and prior to endothelial keratoplasty. Cadaver tissue was obtained from the Lions Gift of Sight Eye Bank (St. Paul, MN) following informed consent by donor’s family or next of kin. Primary cultures of HCECs were prepared from Descemet’s membrane dissected from normal cadaver eyes (n = 10 from 8 individuals; Table 1) or from patients with FECD (n = 7 from 7 patients; Table 1) collected at the time of endothelial keratoplasty. Following collection, tissues were stored in Optisol GS (Bausch & Lomb, Bridgewater, NJ) at 4˚C. For initiating primary cultures, tissues were placed in Opti-MEM (Gibco, Waltham MA) with 8% fetal bovine serum (FBS; Gibco) overnight at 37°C, dissociated with 0.02% EDTA (Sigma, St Louis, MO) in PBS for 60 min at 37°C, and plated in a single well of a 6-well collagen IV-coated plate (Corning, Tewksbury, MA) containing Joyce’s medium [Opti-MEM, 8% FBS, 200 mg/ml CaCl_2_ (Sigma), 0.08% chondroitin sulfate (Sigma), 20 μg/ml ascorbic acid (Sigma), bovine pituitary extract 100 μg/ml (Gibco), 5 ng/ml epidermal growth factor (Millipore, St. Louis, MO), 50 μg/ml gentamicin (Sigma), and 1X antibiotic/antimycotic solution (Invitrogen, Waltham, MA)] [22]. HCECs were allowed to proliferate for 1–4 weeks with media changes every 3–4 days. When cells became 70–80% confluent, cells were passaged. Briefly, cells were rinsed with phosphate buffered saline (PBS, Gibco), incubated in 1X trypsin for 3–5 minutes, dislodged from the plate, and centrifuged for 5 min at 300xg. Supernatant was removed and the cell pellet was resuspended in Joyce’s media and replated at a ratio of 1:3. HCECs were grown to confluence in proliferation medium (defined below) and then incubated in maturation medium (defined below) for 12 days prior to experimentation.

**Table 1:** Demographics and cell line use in experiments.

Normal (cadaver)	Passage*	Age	Sex	Experiment 1	Experiment 2	Experiment 3	Experiment 4	Immortalized
Norm1	3	16	M	X	X		X	
Norm2	3	64	F		X		X	
Norm3	4	64	F		X			
Norm4	4	70	F		X			
Norm5	4	75	F		X			
Norm6	4	9	M			X	X	X
Norm7	2	75	F			X	X	
Norm8	3	61	F			X	X	
Norm9	3	29	F				X	
Norm10	3	29	F				X	
FECD (DMEK)								
FECD1	4	62	F				X	
FECD2	4	71	M				X	
FECD3	4	71	F				X	
FECD4	3	80	F				X	
FECD5	4	65	F				X	
FECD6	3	66	F				X	
FECD7	3	75	M				X	

*Passage number at time of experiment.

Experiment 1: A single HCEC line was used to test the effect of 12 combinations of proliferation and maturation media.

Experiment 2: Additional HCEC lines were tested using Joyce’s proliferation media and different maturation conditions.

Experiment 3: Three HCEC lines were tested using Joyce’s proliferation media and 50% AH as a maturation media.

Experiment 4: Normal and FECD-derived HCEC lines were tested using Joyce’s proliferation media and maturation media with a range from 0–5% FBS content.

#### Proliferation media

Three different proliferation media were used in this study: Joyce’s (defined above), M4 [10,16], and Bartakova (referred to as proliferation media in Bartakova et al) [17]. M4 medium consists of a 50:50 mix of F12 and M199 basal media (Gibco), 5% FBS, 20 μg/ml ascorbic acid, 1X insulin-transferrin-selenium (ITS) supplement (Sigma), 10 ng/ml basic fibroblast growth factor (R&D Systems, Minneapolis, MN), and 1X antibiotic/antimycotic. Bartakova medium consists of Opti-MEM, 8% FBS, 200 mg/ml CaCl_2_, 0.08% chondroitin sulfate, 20 μg/ml ascorbic acid, 100 μg/ml bovine pituitary extract, 5 ng/ml epidermal growth factor, 20 ng/ml human nerve growth factor (Peprotech, Cranbury, NJ), 50 μg/ml gentamicin, and 1X antibiotic/antimycotic solution.

#### Maturation media

Three different maturation media were evaluated: F99 [23], Stabilization 1 [17], and M5 [16]. F99 maturation medium consists of a 50:50 mix of F12 and M199 basal media, 5% FBS, 20 μg/ml ascorbic acid, 1X ITS supplement, and 1X antibiotic/antimycotic. Stabilization 1 maturation medium consists of human endothelial serum free media (endothelial-SFM; Gibco), 4% FBS, 50 μg/ml gentamicin, and 1X antibiotic/antimycotic solution. M5 maturation medium consists of human endothelial-SFM, 5% FBS and 1X antibiotic/antimycotic.

#### Cell proliferation

Following expansion to confluence in proliferation media, HCECs were either maintained in proliferation media (Joyce, M4, Bartakova) or switched to maturation media (F99, Stabilization 1, M5) for 12 days (0–12). A single image (20X) of cells per condition were obtained on days 0, 2, 5, 7 and 12 using a Nikon Eclipse Ti microscope (Melville, NY). Manual cell counting was performed on each image and graphed to determine the effect on cell proliferation.

### Establishment of immortalized HCEC lines

HCECs obtained from the cadaver eye of a 9-year old male were expanded in culture using Joyce’s medium. Passage 2 cells were transduced at the time of plating with VSVG-pseudotyped retroviral vector (pBABE, Addgene, Watertown, MA) encoding the human telomerase reverse transcriptase (hTERT) gene and a puromycin resistance element in the presence of polybrene (0.8 μl/mL, Santa Cruz, Dallas, TX). Transduced HCECs were maintained in Joyce’s medium with puromycin (Sigma) selection pressure (0.75 μg/mL) over three passages. Remaining cells were allowed to expand to approximately 70% confluence where they were isolated and split 1:3 for experimental purposes.

### Flow cytometry

HCECs were grown to confluence in selected proliferation media and cultured for 12 days in corresponding maturation media prior to flow cytometry analysis. All HCECs in each treatment were trypsinized, washed in BD stain buffer for 5 min (BD Biosciences, Franklin Lakes, NJ), and centrifuged at 300xg for 5 minutes. Cells were incubated with fluorescent-conjugated antibodies against CD56, CD73, and CD166 (BD Biosciences) at 4°C for 20 minutes in a container to limit light exposure. Labelled cells were washed three times for 5 min each in BD stain buffer, incubated with 1% formalin (Fisher, Waltham, MA) in BD stain buffer, and analyzed on a BD FACSCanto flow cytometer, collecting data from at least 20,000 cells per treatment. Mean fluorescent intensity (MFI) of live-gated cells was calculated for each marker and compared to other markers to produce a ratio.

### Immunohistochemistry

HCECs were grown to confluence in Joyce’s medium and subsequently cultured for 12 days in selected maturation media. Confluent HCECs grown on collagen IV-coated chamber slides (Ibidi, Gräfelfing, Germany) were fixed with 4% formalin in PBS at room temperature for 10 minutes and washed twice in PBS (5 min each) followed by incubation in methanol at -20°C for 10 minutes. Human corneal tissue dissected from human donor eyes or obtained from patients undergoing endothelial keratoplasty were stored in Optisol GS for ≤24 hours. Tissues were placed in a 24-well plate (Corning), rinsed in PBS, fixed with 1% formalin in PBS at room temperature for 2 minutes, washed once in PBS (2 min), incubated in acetone at -20°C for 5 minutes, and washed three times in 1% DMSO and 5% dextran in PBS (10 min each). Following fixation, tissues (or HCECs) were blocked with 3% bovine serum albumin (BSA, Sigma) in PBS for 30 min at room temperature and incubated at 4˚C overnight with primary antibodies to CD56 (1:100; Novus Biologicals, Littleton, CO) or CD73 (1:50; BD Biosciences) in 0.3% BSA in PBS. Samples were washed three times (5 min each) in PBS and incubated with fluorescence-conjugated secondary antibodies (1:1000; Invitrogen) for 60 min at room temperature in a container that limited light exposure. Following three washes with PBS (5 min each), samples were mounted and counterstained on a glass slide with Vectashield containing DAPI (Vectorlabs, Burlingame, CA). Samples were imaged on a Zeiss LSM780 confocal microscope (Zeiss, Oberkochen, Germany).

## Results

### Comparison of HCEC growth and maturation media

**Fig 1:**
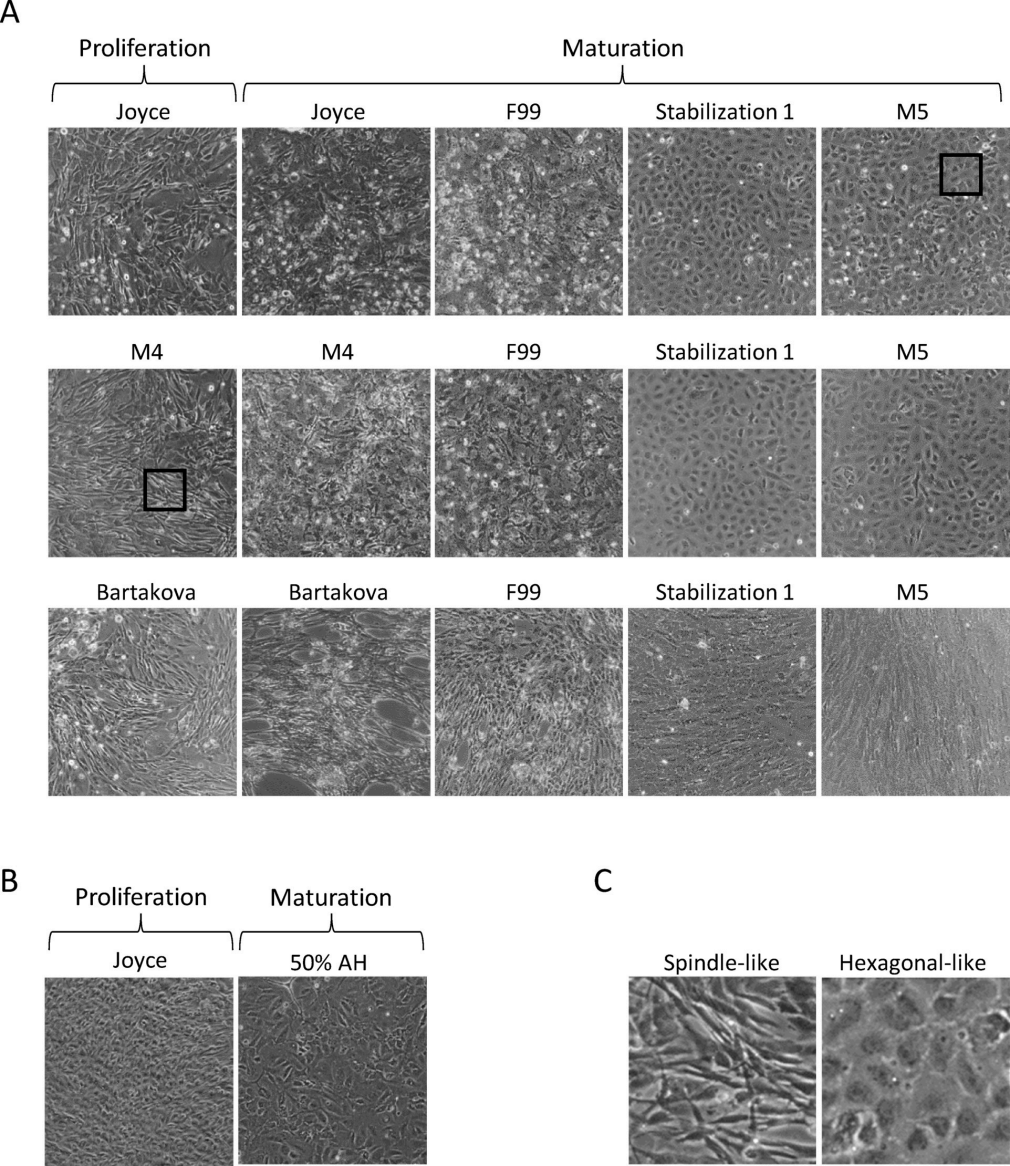
HCECs cultured in various proliferation and maturation conditions. A) HCECs from a 16 year old male donor with no corneal disease were cultured in Joyce’s medium, M4 medium, or Bartakova medium). Upon confluence, media was replaced with various maturation medias. Images were taken of cells at confluence following incubation in proliferation media (Proliferation) and after 12 days in maturation media (Maturation). B) HCECs proliferated in Joyce’s media with images taken at confluence (Proliferation) and following 12 days (Maturation) in human endothelial-SFM supplemented with 50% human aqueous humor (AH). Experiment was performed with three independent HCEC lines. C) Expanded insets (black boxes) from M4 proliferation only image (A) and Joyce with M5 maturation image (A) to illustrate spindle-like vs hexagonal-like HCEC morphologies.

To identify in vitro conditions that consistently produce high quality primary HCEC cultures, we examined several proliferation and maturation media that have been reported to maintain HCEC in vivo characteristics. Using a single eye obtained from a young donor (16-year old male), corneal endothelial cells were isolated and initially placed in culture with Joyce’s medium. Once cells began to proliferate, cells were either maintained in Joyce’s medium or switched to M4 or Bartakova proliferation media. HCECs proliferated at a faster rate in Bartakova medium, reaching confluency in 3 days (Fig 1A), while cells in Joyce’s or M4 media reached similar confluency in 5 days. Regardless of the proliferation medium used, all groups showed flat adherent cells with varying cell morphology (Fig 1A and 1C).

**Fig 2:**
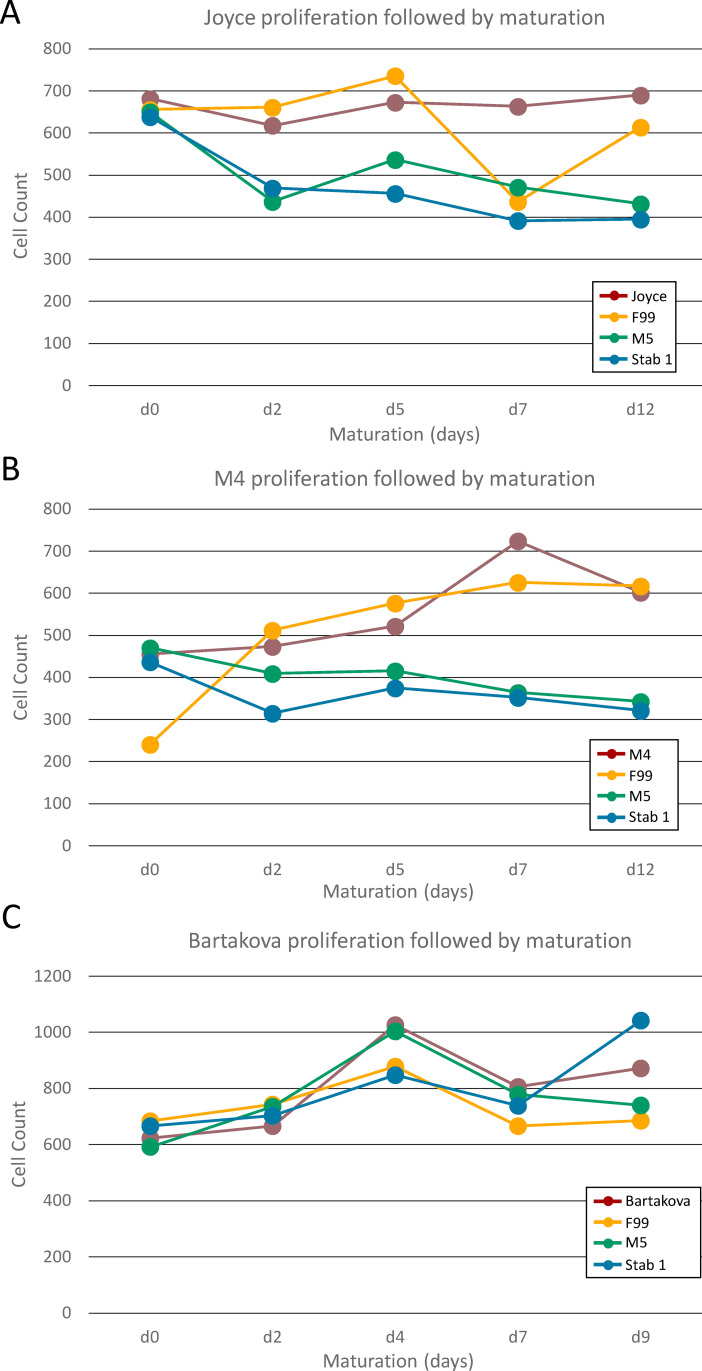
HCEC proliferation in maturation media. HCECs maintained in Joyce (A), M4 (B) or Bartakova (C) proliferation media showed a slight increase in cell density during the maturation phase (day 0–12). Similar results were observed when cells were switched from proliferation media to F99 maturation media. (A, B) In contrast, HCECs incubated in Stabilization 1 (Stab 1) or M5 maturation media for 12 days showed a slight decrease in cell density indicating that cells were not in a proliferative phase. (C) HCECs originally expanded in Bartakova proliferation media either maintained or continued to proliferate in F99, Stabilization 1, or M5 maturation media.

To investigate the impact on morphology of single compared to dual media approaches, HCECs that had achieved confluence were either maintained in proliferation media (Joyce, M4, Bartakova) or changed to maturation media (F99, Stabilization 1, M5). After 12 days, HCECs that were maintained in proliferation media showed a slight increase in density (Fig 2), with cells growing over one another in some areas (Fig 1A), indicating a loss of contact inhibition. Cells in Bartakova medium showed some detached cells around day 10 probably due to increased cell proliferation and excessive loss of contact inhibition. Additionally, HCECs grown in proliferation media alone (Joyce, M4 or Bartakova) showed a widely variable morphology within cultures, with many cells appearing fibroblast-like, with spindle or narrow cuboidal shapes.

HCECs grown with dual media (proliferation medium followed by maturation medium) showed a more uniform morphology. Cultures grown in Bartakova medium followed by maturation in Stabilization 1 or M5 media did show some elongated spindle-shaped cells but overall exhibited a more uniform monolayer with cells tending to grow in parallel with one another. HCECs grown in Joyce’s or M4 proliferation media followed by maturation in Stabilization 1 or M5 media had the most uniform, hexagonal-like appearance, reminiscent of in vivo corneal endothelial cells (Fig 1A and 1C). Cultures incubated in Stabilization 1 or M5 media did not show any proliferation, with a slight decrease in cell numbers from day 0 to day 12 (Fig 2).

### Expression of CD56, CD166 and CD73

To correlate morphological appearance with marker expression, HCECs grown in each of the culture conditions described above were assessed by flow cytometry to quantify expression profiles of CD56, CD166 and CD73. Regardless of which proliferation medium was used (Joyce, M4, Bartakova), CD56 was elevated in all maturation conditions (F99, Stabilization 1, M5) compared with HCECs that were maintained in proliferation media alone, with highest expression observed in Stabilization 1 and M5 media when compared to maturation in F99 (Table 2). CD166 expression was similarly elevated in all 3 maturation media compared with proliferation media alone but was not consistently different between maturation conditions. Conversely, CD73 expression was higher in HCECs maintained in proliferation media or matured in F99 medium, and lower in Stabilization 1 and M5 maturation conditions, regardless of initial proliferation media.

**Table 2:** Expression ratios in HCECs following proliferation and maturation in different media.

Proliferation medium	Maturation medium	CD56 (MFI)	CD166 (MFI)	CD73 (MFI)	CD56:CD73	CD166:CD73
Joyce	Joyce	837	491	5768	0.15	0.09
	F99	3023	2301	11441	0.26	0.20
	Stabilization 1	6452	1106	1790	3.60	0.62
	M5	6585	1127	1997	3.30	0.56
M4	M4	1540	632	5425	0.28	0.12
	F99	2030	1079	6867	0.30	0.16
	Stabilization 1	6196	1121	2394	2.59	0.47
	M5	5954	1058	2339	2.55	0.45
Bartakova	Bartakova	154	973	9999	0.02	0.10
	F99	338	1072	9224	0.04	0.12
	Stabilization 1	3827	875	1767	2.17	0.50
	M5	5043	1159	2257	2.23	0.51

MFI; mean fluorescence intensity.

Analysis of all expression phenotypes from cells grown in different culture conditions showed that the ratio of CD56:CD73, calculated from flow cytometry mean fluorescence intensity (MFI), directly correlated with cell morphology. CD56:CD73 values ranged from 0.02 (low CD56, high CD73) in the most spindle-shaped, fibroblast-like cells (where proliferation medium was used throughout the growth and maturation phase) (Fig 1C, representative image) to >2.5 (high CD56, low CD73) in cultures that showed uniform and hexagonal-like-appearing cells (Joyce’s or M4 proliferation media followed by Stabilization 1 or M5 maturation media; Table 2 and Fig 1C, representative image). The ratio of CD166:CD73 showed similar results to CD56:CD73 ratios, with low values (<0.2) obtained in cultures with spindle-shaped cells that were maintained in proliferation media throughout the maturation phase. Higher CD166:CD73 ratios were also seen in cells incubated in Joyce’s or M5 media followed by maturation in Stabilization 1 or M5 media, although these ratios (>0.2) were lower than that observed with CD56:CD73 ratios (>2.5). Both CD56:CD73 and CD166:CD73 ratios were maximal when cells were incubated in Joyce’s media during the proliferation phase and Stabilization 1 or M5 during the maturation phase, which correlated with hexagonal-like HCEC morphologies most similar to corneal endothelial cells in vivo.

### Validation of HCEC culture conditions

To assess whether HCEC cultures established from older patients showed similar morphology and marker expression as the initial young donor, we established 4 additional HCEC lines from human donor eyes aged 64–75 years old (Table 1). Based on results above, we selected Joyce’s as the proliferation medium and used it in combination with all 3 maturation media. Following proliferation in Joyce’s medium and maturation in either F99, Stabilization 1, or M5 maturation medium, cultures showed similar cell morphology to those observed in HCECs from the younger donor. Specifically, cells established from older donors matured in F99 showed non-uniform and spindle shaped cells while cells matured in Stabilization 1 and M5 media had a uniform, hexagonal-like appearance.

Flow cytometry was performed on each culture and corresponding MFI values were recorded (see S1 Table for individual MFI values for each experiment). Average ratios of CD56:CD73 and CD166:CD73 were 0.63 ± 0.54 and 0.48 ± 0.34 when matured in F99, 9.73 ± 3.03 and 3.07 ± 0.55 in Stabilization 1, and 8.34 ± 3.47 and 2.84 ± 0.95 in M5 (Table 3). CD56:CD73 and CD166:CD73 expression ratios were higher (p<0.005) in Stabilization 1 and M5 than in F99 maturation conditions. HCECs maintained in Joyce’s medium alone for proliferation and maturation phases had CD56:CD73 (0.13 ± 0.09) and CD166:CD73 (0.18 ± 0.05) ratios that were significantly lower (p<0.005) compared to HCECs matured in Stabilization 1 or M5 media, and displayed concomitant elongated and non-uniform cell morphology.

**Table 3:** Expression ratios in HCECs following proliferation in Joyce’s medium and maturation in several different media.

Proliferation medium	Maturation medium	Sample size	CD56:CD73	CD166:CD73
Joyce	Joyce	4	0.13 ± 0.09	0.18 ± 0.05
	F99	4	0.63 ± 0.54	0.48 ± 0.34
	Stabilization 1	4	9.73 ± 3.03	3.07 ± 0.55
	M5	4	8.34 ± 3.47	2.84 ± 0.95
	50% AH	3	7.23 ± 1.52	24.17 ± 5.86

AH–human aqueous humor.

### Effect of human aqueous humor on HCECs

To evaluate the effect of human aqueous humor on HCEC characteristics compared to the culture conditions identified above, HCECs (n = 3) were proliferated in Joyce’s medium and matured in human endothelial-SFM containing 50% human aqueous humor. HCEC lines matured in 50% aqueous humor showed variable cell density but all cultures showed a uniform monolayer with hexagonal-like shaped cells (Fig 1B). Average CD56:CD73 and CD166:CD73 ratios were 7.23 ± 1.52 and 24.17 ± 5.86 in cells matured in 50% aqueous humor (Table 3). Similar CD56:CD73 expression ratios were found in HCECs proliferated in Joyce’s medium and matured in either Stabilization 1 or M5 medium (p = 0.55). CD166:CD73 ratios in cells matured in 50% aqueous humor were greater than cells treated with other maturation conditions (F99, Stabilization 1, M5) (p<0.001).

### In vivo expression of CD56 and CD73

**Fig 3:**
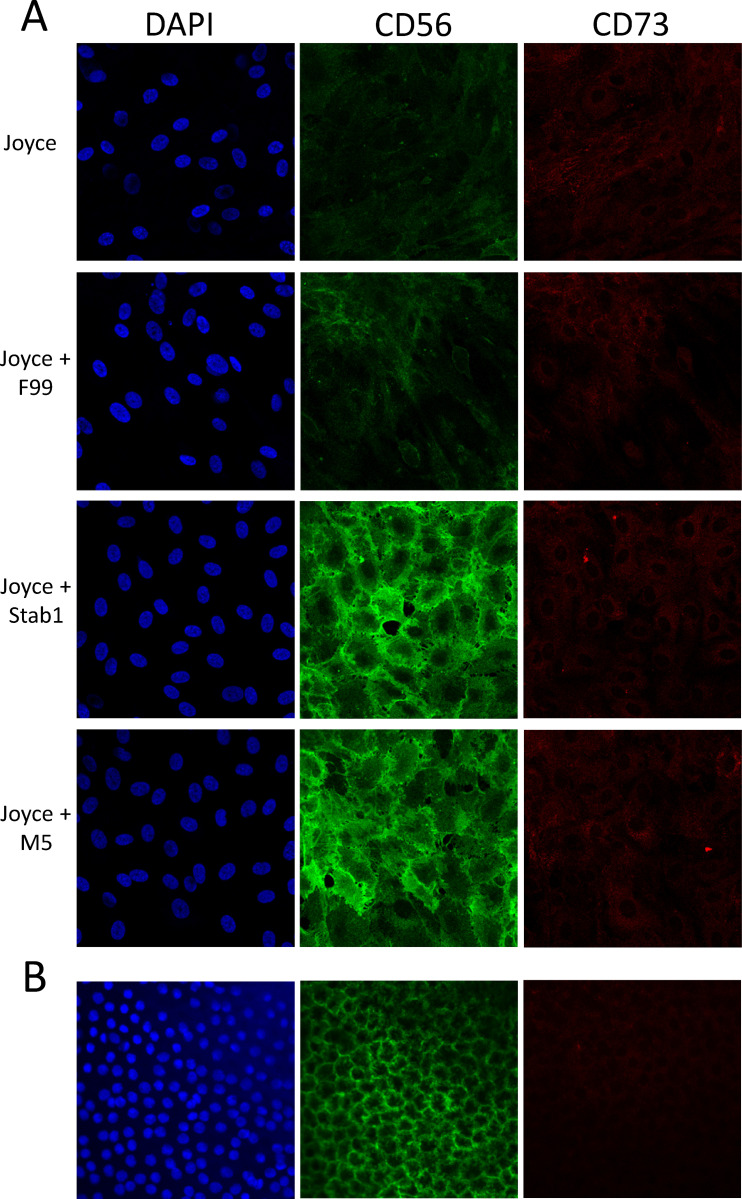
Expression of CD56 and CD73 in HCECs in various culture conditions. A) Representative images of HCECs from donors with no corneal disease, proliferated in Joyce’s medium until confluent and matured in indicated medium for 12 days. HCECs were stained for CD56 (green) and CD73 (red) and counterstained with DAPI (blue) to show nuclei. B) Corneal endothelial tissue from a donor with no corneal disease was stained for CD56 (green) and CD73 (red), and counterstained with DAPI (blue) to show nuclei.

Immunofluorescence imaging of CD56 and CD73 in HCECs provided qualitative confirmation of CD56 and CD73 expression ratios obtained by flow cytometry (Fig 3A). CD56 expression was greatest in cultures matured in Stabilization 1 or M5 medium. In contrast, CD73 expression was low with cells showing minimal staining in the cytoplasm. Similar expression and localization of CD56 and low CD73 expression was observed in human corneal endothelium obtained from a normal human donor (Fig 3B). CD56 was expressed at high levels in contrast to CD73 which was barely visible. Like CD56 in HCECs cultured in Joyce’s medium and matured in either Stabilization 1 or M5, expression of CD56 in tissue was found localized at the cell boundaries. These results further validated the use of the CD56:CD73 expression ratio as a measure to predict in vivo-like HCEC morphologic characteristics.

### Evaluation of low mitogen containing maturation media on HCECs

**Fig 4:**
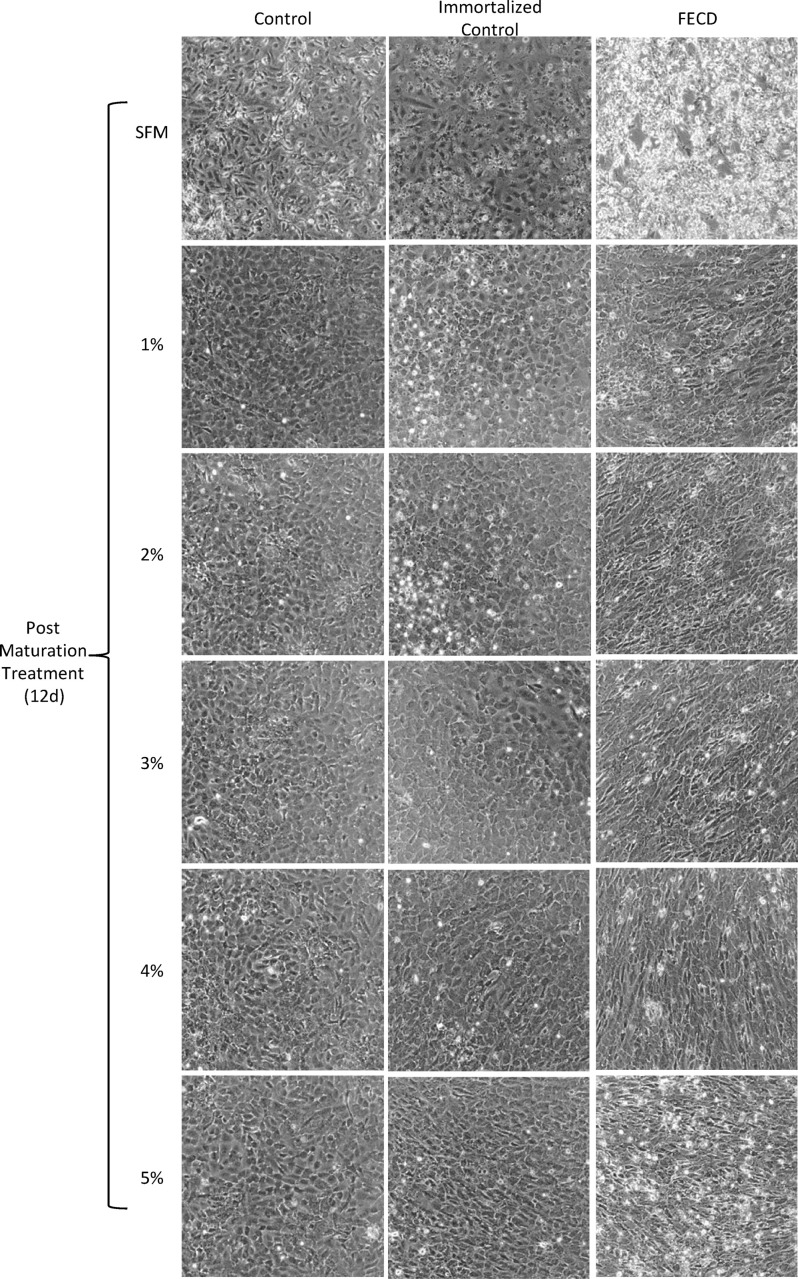
Morphology of normal, immortalized, and FECD-HCECs after maturation with different concentrations of FBS. Representative images of confluent HCECs following proliferation in Joyce’s medium and maturation in human endothelial-SFM supplemented with 0–5% FBS. HCECs from a 9 year old male donor (Norm6) with no corneal disease are on the left, HCECs from same donor after hTERT immortalization are center, and FECD-HCECs are on the right.

**Fig 5:**
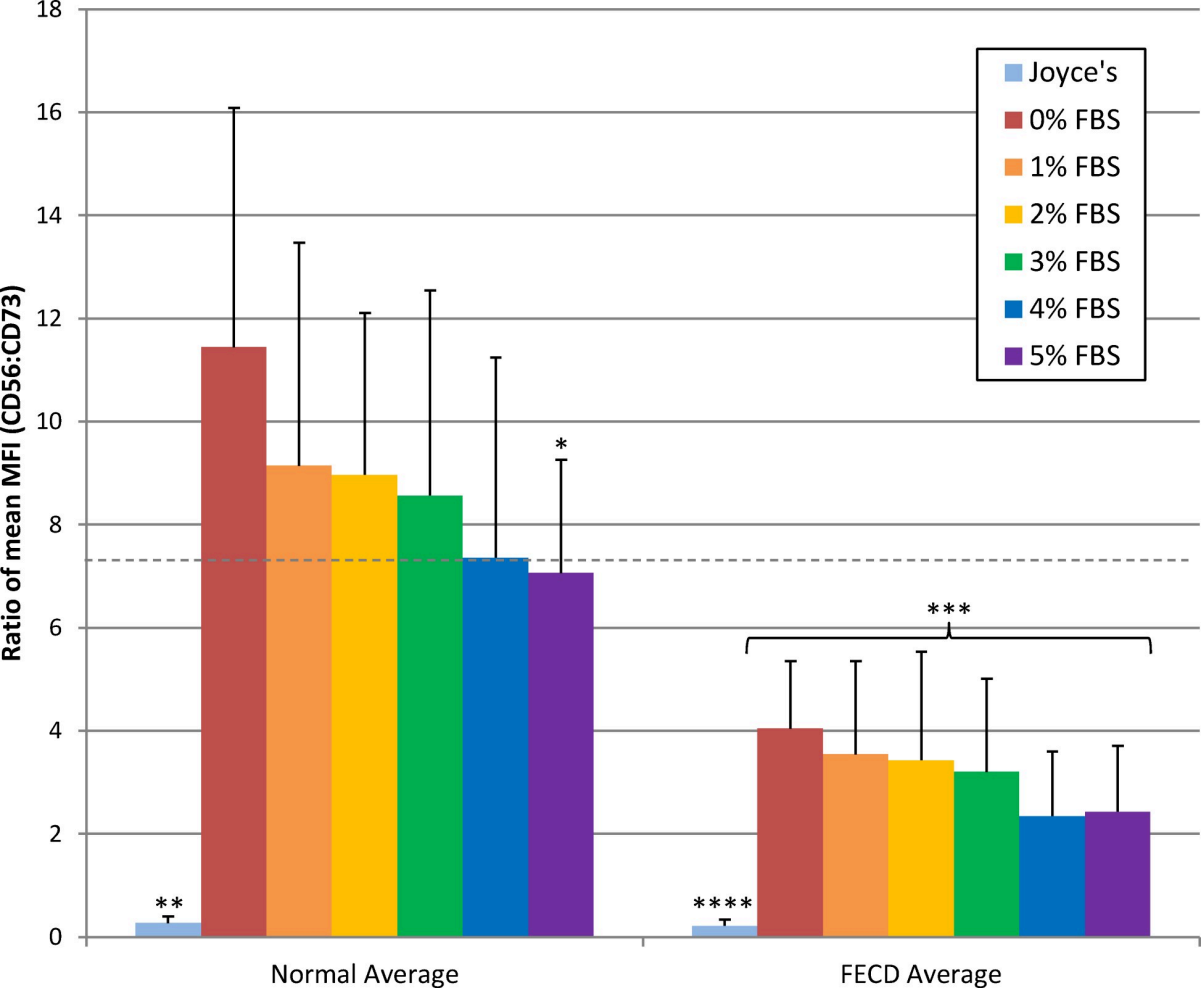
Expression ratios of CD56:CD73 from normal and FECD-HCECs matured in various concentrations of FBS. HCECs were stained for CD56 and CD73 and analyzed by flow cytometry. After gating for live cells, the mean fluorescent intensity (MFI) of CD56 and CD73 for each line was used to generate the CD56:CD73 ratio (see S1 Table for individual MFI values). Dotted line denotes value of CD56:CD73 ratio in normal HCECs matured in 50% aqueous humor. Normal HCECs, n = 7; FECD-derived HCECs, n = 7. *p = 0.04 to normal 0%; **p<0.0005 Joyce’s to all normal maturation medias; ***p<0.01 FECD-HCEC maturation medias to respective normal HCECs; ****p<0.01 Joyce’s to all FECD-HCEC maturation medias.

The maturation media that produced the most consistent in vivo-like corneal endothelial cell morphology were Stabilization 1 and M5, which differ only in their concentration of FBS, with Stabilization 1 medium containing 4% FBS and M5 containing 5% FBS. Therefore, we decided to analyze the effects of FBS concentration on HCEC cell marker expression. For this, HCEC lines (n = 7) were expanded in Joyce’s medium and matured in human endothelial-SFM supplemented with 0, 1, 2, 3, 4 or 5% FBS for 12 days. While all culture conditions showed mostly uniform monolayers containing hexagonal-like shaped cells, cultures in low serum concentrations (1–3%) tended to be more consistent regarding cell appearance across different HCEC lines (Fig 4). Analysis of expression levels showed average CD56:CD73 and CD166:CD73 ratios of 11.45 ± 4.65 and 15.45 ± 20.07 in 0%, 9.14 ± 4.33 and 14.94 ± 19.95 in 1%, 8.97 ± 3.15 and 17.19 ± 21.59 in 2%, 8.57 ± 3.98 and 17.35 ± 22.50 in 3%, 7.36 ± 3.88 and 18.17 ± 24.25 in 4%, and 7.06 ± 2.20 and 15.02 ± 19.26 in 5% FBS (Fig 5). The CD56:CD73 expression ratio suggests that CD56 expression is highest in serum-free maturation media and slightly decreases as serum concentration increases. However, some HCECs maintained in 0% FBS started to release from the plate the longer they remained in culture, suggesting unhealthy cells, presumably due to lack of nutrients and growth factors supplied by the serum. CD56:CD73 ratios for all serum concentrations were significantly different than the same cells cultured for the same duration in Joyce’s medium (CD56:CD73 of 0.27 ± 0.13; p<0.0005) (Fig 5). However, only the 0% and 5% FBS treatments had CD56:CD73 expression ratios that were significantly different when compared (p = 0.04) to each other. CD166:CD73 ratios were elevated in all serum concentrations, but showed dramatic variability between cell lines, reducing the utility of this measure as a predictive indicator.

### Analysis of culture conditions on immortalized HCECs

**Fig 6:**
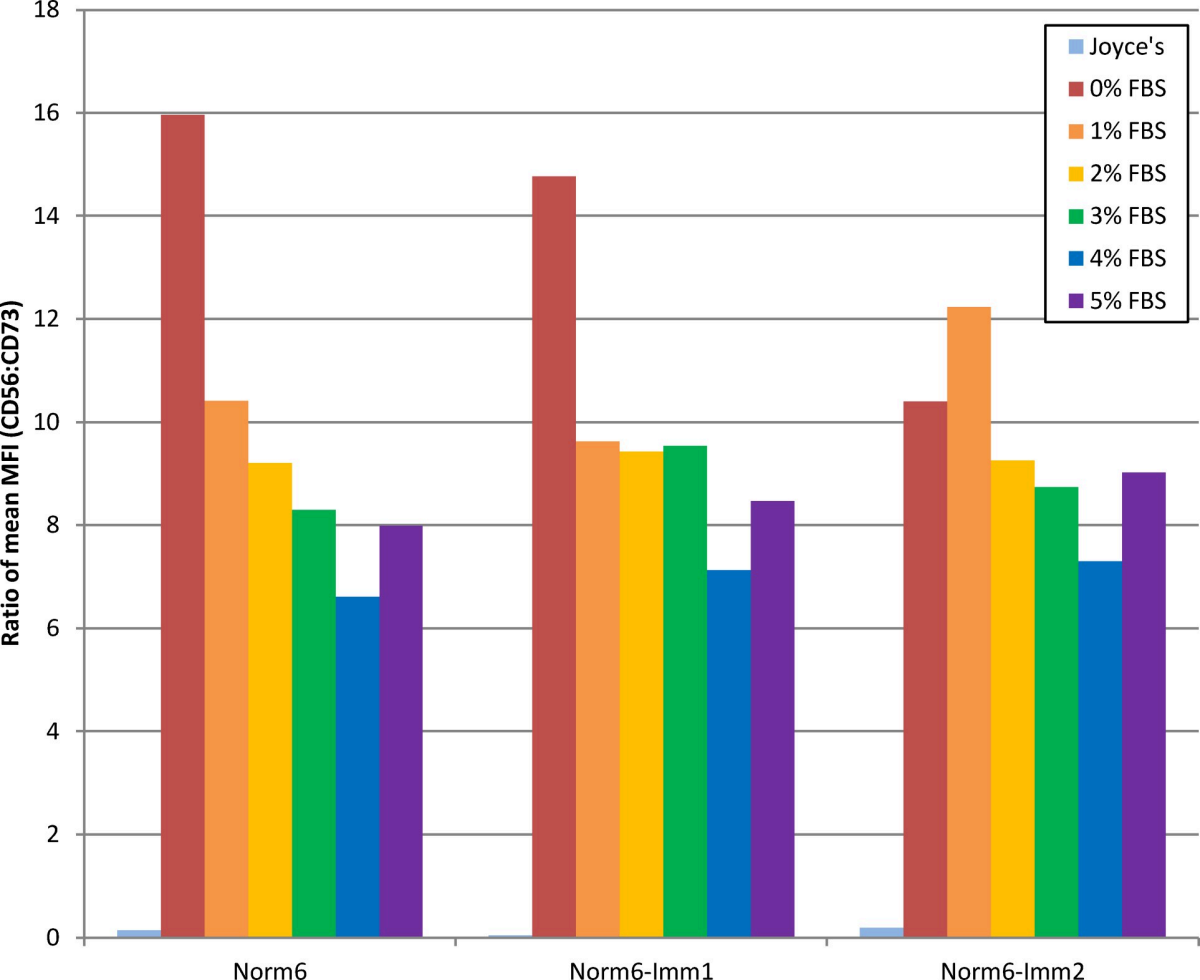
Expression ratios of CD56 and CD73 from normal and hTERT immortalized HCECs matured in various concentrations of FBS. Two independent immortalized cell lines (Norm6-Imm1 and Norm6-Imm2) were established from Norm6. CD56 and CD73 expression was analyzed by flow cytometry and the mean fluorescent intensity (MFI) of CD56 and CD73 for each line was used to generate the CD56:CD73 ratio. S1 Table contains individual MFI values.

In addition to primary HCECs, immortalized HCEC lines have been developed and utilized in biochemical studies to understand corneal endothelial molecular and physiological characteristics [24–26]. To assess morphology and marker expression ratios in immortalized normal HCECs, two cell lines were independently established from a single parental cell line (Norm6) by retroviral transduction with hTERT. Comparison of the immortalized HCEC lines to their parental primary cell line when expanded and matured in the same media conditions (proliferated in Joyce’s medium and matured in 0–5% FBS) showed similar CD56:CD73 expression ratios, with an average percent difference of only 10.6% (Fig 6). Morphology as viewed by light microscopy did not show any consistent differences between parental and immortalized cell lines in any media treatments (Fig 4), indicating minimal effect of hTERT immortalization on the parental line with reference to cell morphology and CD56 and CD73 expression.

### Analysis of culture conditions on HCECs-derived from FECD patients

Having established proliferation and maturation media sufficient to maintain normal primary and immortalized HCECs in culture, we assessed the morphology and marker expression ratios in HCEC cultures established from patients with FECD (herein referred to as FECD-HCECs). Performing the same experiment as outlined for normal and immortalized HCECs (proliferation initiated in Joyce’s medium and matured in 0–5% FBS), FECD-HCECs (n = 7) showed greater variation in density and morphology within cell lines and between independent cultures. Maturation treatments resulted in cells with regular, hexagonal-like morphology, although there was more variation in cell shape compared to matured populations of normal HCECs. Also, FECD-HCECs had a greater tendency to release from the plate at 0% and 1% FBS treatments (Fig 4). CD56:CD73 and CD166:CD73 ratios of 4.05 ± 1.31 and 3.89 ± 3.50 in 0%, 3.55 ± 1.81 and 3.84 ± 3.81 in 1%, 3.43 ± 2.31 and 4.29 ± 4.94 in 2%, 3.20 ± 1.82 and 4.82 ± 5.40 in 3%, 2.35 ± 1.26 and 4.30 ± 5.26 in 4%, and 2.43 ± 1.29 and 4.20 ± 5.13 in 5% FBS were observed in FECD-HCECs (Fig 5). When proliferated and maintained in Joyce’s medium alone, FECD-HCECs had a significantly different CD56:CD73 ratio (0.22 ± 0.13; p<0.002) from all FECD maturation treatments (0–5% FBS). Interestingly, when normal, immortalized, or FECD-HCECs were maintained in Joyce’s medium throughout the proliferation and maturation phases, the cultures showed no significant differences in CD56:CD73 expression ratios (p = 0.34).

**Fig 7:**
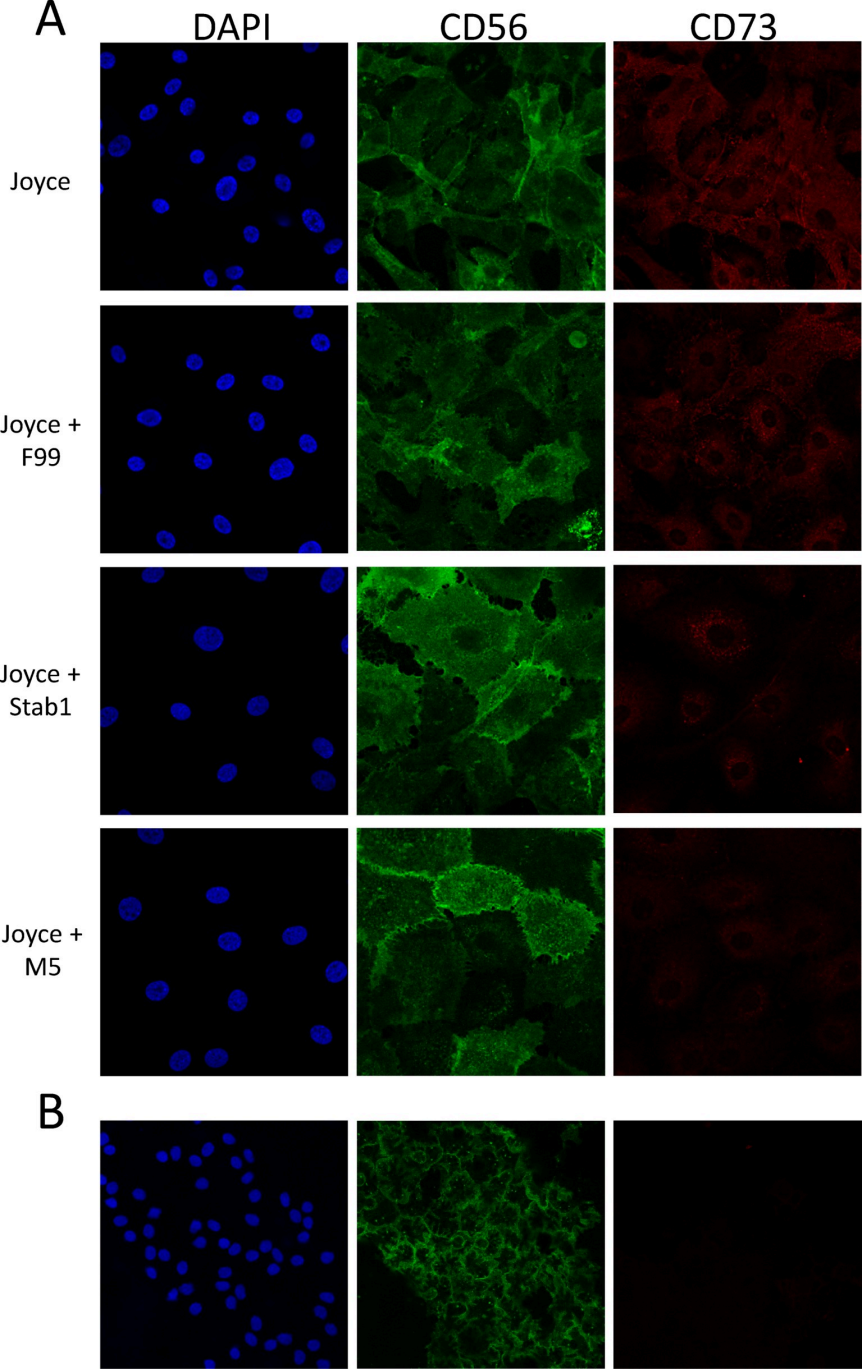
Expression of CD56 and CD73 in FECD-HCECs in various culture conditions. A) Representative images of FECD-HCECs proliferated in Joyce’s medium until confluent and changed to indicated maturation media for 12 days. Cells were stained for CD56 (green) and CD73 (red) and counterstained with DAPI (blue) to show nuclei. B) FECD patient derived CE tissue stained for CD56 (green) and CD73 (red), and counterstained with DAPI (blue) to show nuclei.

The CD56:CD73 expression ratios in FECD-HCECs were 2-3-fold less than ratios in normal HCECs after maturation in all 0–5% FBS conditions (p≤0.01). This was due to both lower expression of CD56 (FECD-HCEC expression averaged 66% of normal HCECs in matched maturation conditions) and higher expression of CD73 (FECD-HCEC expression averaged 175% of normal HCEC in matched maturation conditions). However, considering all HCEC types and culture conditions, a CD56:CD73 ratio >2.5 can be considered correlative and predictive of in vivo-like corneal endothelial characteristics by HCECs. To visually confirm lower CD56:CD73 expression ratios, immunohistochemistry was performed on FECD-HCEC cultures and corneal endothelial tissue isolated from a FECD patient undergoing endothelial keratoplasty. Staining for CD56 after growth in maturation conditions was notably less intense in FECD derived cell lines and FECD CE tissue (Fig 7) compared to normal (Fig 3). In tissue, nuclear staining revealed irregular cell spacing in FECD compared to normal CE, and that disorganization was mirrored in the localization of CD56 expression in FECD-HCECs, which closely followed cell borders.

## Discussion

Maintaining in vivo characteristics in primary HCEC cultures is a necessary goal for researchers using this model system to obtain relevant data to understand biological characteristics and molecular mechanisms in corneal dystrophies. Our data shows that use of a dual media approach that includes a low-mitogen-containing maturation medium, is essential to identify significantly high CD56:CD73 and CD166:CD73 expression ratios. These ratios coincide with a regular and reproducible HCEC morphology that is phenotypically reminiscent of corneal endothelial cells in vivo.

In this study, we evaluated the expansion of HCECs in three proliferation media (Joyce, M4, Bartakova) that differed slightly in their basal media compositions, growth factor contents, and FBS concentrations. HCECs were found to proliferate well in each medium. Bartakova medium resulted in the most prolific dividing cells and showed the lowest CD56:CD73 and CD166:CD73 expression ratio (0.1–0.2) consistent with their elongated and fibroblast-like appearance. Incubation with either Joyce’s or M4 media resulted in a slower proliferation rate than Bartakova media (5 days to reach confluence), but still produced low CD56:CD73 and CD166:CD73 expression ratios. The only difference between Bartakova and Joyce’s media is the inclusion of nerve growth factor in the former, suggesting that this growth factor may have been involved in stimulating a higher proliferative rate in HCECs. The M4 proliferation medium differed from Joyce’s and Bartakova in its basal media (F12/M199) and a lower FBS concentration (5%). HCECs grown and maintained in M4 had the highest CD56 expression of any proliferation media alone, but also retained higher CD73 expression when cells were subsequently matured in other media. Joyce’s medium showed the most consistent proliferation potential and the highest CD56:CD73 and CD166:CD73 expression ratios when subsequently matured in low mitogen containing media. However, HCECs maintained in Bartakova, M4 or Joyce’s proliferation media (single medium culture) did not produce cultures reminiscent of corneal endothelial cells in vivo, and while there were differences in CD56:CD73 and CD166:CD73 expression ratios, these were consistently low, indicating a cell population with a mixed endothelial/fibroblast-like phenotype.

HCECs that were initially cultured in M4 and Joyce’s proliferation media and followed with maturation in low mitogen containing medias (F99, Stabilization 1, M5) produced cultures that were more representative of corneal endothelial cells. The F99 medium, which is similar to M4 proliferation medium but without basic fibroblast growth factor, produced HCEC cultures with elevated CD73 expression and cells with less hexagonal-like appearing cells when compared to cultures matured in Stabilization 1 or M5 media. Stabilization 1 and M5 media used human endothelial-SFM basal media supplemented with 4% or 5% FBS respectively, and no other supplementation. Use of maturation media with lower FBS concentrations (1–3%) produced higher CD56:CD73 expression ratios and HCEC cultures with cell morphology reminiscent of corneal endothelial cells in vivo. Use of no FBS or 1% FBS showed the highest CD56:CD73 ratios but cells tended to detach from the substrate. HCEC cultures that were incubated in human endothelial-SFM media containing 2% FBS showed consistently high CD56:CD73 expression ratios and cell morphology similar to HCEC cultures matured in 50% aqueous humor. Based on results from this study, use of a dual media approach that includes proliferation in Joyce’s medium followed by maturation in endothelial-SFM containing 2% FBS is now routinely used in our laboratory to establish HCEC cultures.

Immunostaining of HCEC cultures and corneal endothelium tissue reinforced the low-mitogen maturation approach, resulting in CD56 and CD73 staining patterns in culture that were similar to those in tissue. CD56 was clearly present at varying degrees in HCECs at levels that were consistent with our flow cytometry results, i.e. lower in cultures with proliferation media and higher in cultures after maturation with low-mitogen media. CD56 immunolabeling not only showed higher expression in low-mitogen maturation media, but also showed increased expression at the cell boundaries, similar to that observed in normal corneal endothelial tissue. In contrast, CD73 expression, while universally low, was higher in Joyce’s medium and barely detectable in low-mitogen maturation media.

Immortalization of HCECs has been used to produce a consistent cell line with morphological and gene expression similarities to primary HCECs for experiments requiring large number of cells or multiple passages. Having a single immortalized cell line can remove the variation between independent cell lines and alleviate the need for fresh tissue. As with any immortalization, it is important to monitor for dedifferentiation or loss of primary cell characteristics so that the immortalized model maintains cell characteristics similar to the primary cell type. Immortalized HCECs developed by transduction with SV40 large T antigen or HPV16 E6/E7 have shown expression of several corneal endothelial markers that were maintained from primary to immortalized cells [24,26]. Our analysis of immortalized cell lines following hTERT transduction, performed at 5–7 passages beyond immortalization, suggests that when grown and matured using a dual media culture system, these cells will maintain morphological and expression levels consistent with primary HCECs.

Establishing appropriate proliferation and maturation conditions for normal HCECs is essential as it is only then that appropriate conclusions can be drawn from experimentation. This is of particular importance when evaluating FECD-HCECs. Use of a culture system that potentiates normal growth can be used on diseased cells to identify abnormal cellular mechanisms associated with disease pathology. Reduced antioxidant levels and susceptibility to oxidative damage, endoplasmic reticulum stress, cellular senescence, and inducible cell proliferation with Rho-Kinase inhibitors have all been linked to FECD pathogenesis through investigation of primary FECD-HCECs [27–31]. Application of the CD56:CD73 ratio to FECD-HCEC cultures yielded somewhat lower CD56:CD73 ratios when cultured in proliferation and maturation conditions established in normal HCECs, and FECD-HCECs showed less cell uniformity and more variability in their cell shapes consistent with a lower CD56:CD73 ratio. It is possible that incubation, regardless how the medium is optimized, may select healthier cells potentially biasing the FECD-derived cultures. While this may be true, the CD56:CD73 ratio was still lower than normal HCECs suggesting differences in these cells. Until aqueous humor from normal and FECD patients reveals unrecognized growth factors or cytokines that alter corneal endothelium, it will be difficult to develop a specific medium for FECD-HCECs. Understanding the degree of association of the CD56:CD73 ratio with progression of corneal pathologies will be required to help standardize proliferation and maturation conditions in future studies.

The change in ratio in FECD-HCECs was driven by reduced expression of CD56 and elevated expression of CD73. This was confirmed by immunohistochemical staining of FECD-HCEC and FECD explant tissue which showed significant disruption of monolayer organization, including less CD56 localization at the cell boundaries. It is interesting to note that patients with FECD have reduced cell density which is thought to be due to changes in cell/cell and cell/extracellular matrix adhesion. CD56 is an adhesion molecule and its lower expression in FECD cells may correlate to reduced adhesion, potentially resulting in cell loss. Similarly, the higher levels of CD73 may be a useful marker for FECD-HCECs as these cultures tend to have a less uniform hexagonal-like appearance with more cells showing fibroblast-like morphology. In its non-enzymatic form, CD73 alters adhesion to several extracellular molecules (e.g. laminin and fibronectin), enabling increased migration [32,33]. The lower CD56 levels and higher CD73 levels found in FECD cells compared to normal corneal endothelial cells may help to influence FECD-like behavior. It is important to note that had we only made a comparison between marker expression using proliferation media (Joyce’s), we would not have detected any difference between marker expression in normal and FECD-HCEC lines. It was only after maturation in low-mitogen conditions that expression differences were consistent, highlighting the importance of a dual media system to optimize HCEC cultures.

While our study tested the effect of a single compared to a dual media system to examine the effects on CD expression and morphology, we did not perform functional studies nor did we compare transcriptome expression between the different conditions. While these were beyond the scope of the present study, recently published data do conform in principle with our study. Frausto et al has reported in a similar dual media culture system that HCEC barrier and pump functions are more robust compared to a single media culture system [18]. Similarly, HCECs in a dual media system recapitulated a 97 gene in vivo expression profile more closely than HCECs grown in single medium.

In summary, we have examined several media variations for development of primary HCEC cultures and identified an optimal dual media system using Joyce’s medium that promotes HCEC proliferation followed by incubation in a low-mitogen containing maturation media (human endothelial-SFM with 2% FBS) to produce regular, hexagonal-like monolayers mimicking corneal endothelial morphology in vivo. These HCECs also showed a high CD56:CD73 expression ratio, indicating a strong correlation between in vivo morphology and expression profile of these markers. Overall, these results help standardize culture conditions of HCECs and aid in optimizing an in vitro model system for studying pathophysiology of corneal endothelial dystrophies like FECD.

## Supporting information

S1 Table Mean fluorescent intensity (MFI) of live-gated cells for individual cell lines and media conditions. (XLSX)
